# Therapeutics

**Published:** 1875-12

**Authors:** 


### IV. THERAPEUTICS.

1. *Researches on the Absorption and Elimination of Iodine.* ED. WELANDER (*Allg. Med. Central Zeitung*, 1875. No. 77.)

During the past three years the author has been experimenting with iodine in the hospital at Stockholm; he administered it per os, per anum, hypodermically, upon the wounded and unwounded skin. It was used in the form of the iodides of potassium, iron and mercury, and given in solution, powders, pills and suppositories. He generally used the urine as a test-object; sometimes he also tested the saliva, and the milk whenever he had an opportunity for so doing. Iodine taken through the mouth appeared more quickly and abundantly in the urine the more soluble a preparation was chosen; given in pills, each containing one grain of an iodide, the iodine was found in the urine after 15 minutes when iodide of potassium, after 3 to 4 hours when iod. of iron, and after 4 to 5 hours faintly when iod. of mercury was chosen. The resorption is still more rapid with solutions of the iodides; after one-sixth of a grain of iod. potass. was taken, the urine showed the reaction of iodine for the next 12 to 24 hours; and if a person had taken 15 grains of iod. potass. daily for some time, the iodine-reaction of the urine could be shown 3 to 4 days after discontinuance of the medicament. The same results were obtained when the preparations were administered per anum; this fact is important, as there may be cases in which the remedy cannot be given through the stomach; then the rectum may be substituted, and the effect of the remedy obtained nevertheless. In three cases iod. potass. was introduced hypodermically and copiously appeared in the urine within 30 minutes, and continued for 5 hours. The absorption through wounds or granulating surfaces was tried in four cases and found very active. But iodine applied to the unwounded skin (in ointment or solution) could not be traced in the urine. The saliva whenever tested, contained iodine, and so did the milk of the mother and the urine of the infant. The amount of iodine in the milk and that in the infant's urine were always proportionate, but in the latter it appeared later and lasted longer. So in one case when the mother had taken ten grains of potass. iod., iodine was discovered in the milk for 30 hours and in the infant's urine for 58 hours.

2. *Purgatives.* T. LAUDER BRUNTON, M.D. (*The Practitioner.*)

"Purgatives act by stimulating the secretion of fluid from the intestines, as well as by increasing peristaltic action. They prove useful in many ways. They hurry food out of the alimentary canal and thus lessen the injurious effects of overeating. By expelling irritating substances from the intestine they arrest diarrhœa, and remove headache and other pains, caused either by the abdominal irritation or by the absorption of poisonous matters produced by imperfect digestion and decomposition of food. They relieve biliousness by removing bile, and are most efficient aids in the treatment of chronic poisoning by lead, mercury or other metals. It is probable that pepsin and pancreatic ferment are absorbed from the intestine and circulate in the blood, where the latter assists in the production of animal heat. They are then secreted anew by the stomach and pancreas, and do their work again. Purgatives lessen their quantity as well as that of the bile; they may thus be useful in fevers, but they injure old and feeble persons, both by diminishing their calorific power and impairing their digestion. They relieve inflammation by lowering the blood pressure and thus diminishing congestion; and they prove beneficial in dropsies both by abstracting water from the blood and diminishing congestion in the kidneys."

3. *Cantharidal Strangury.* STARR. (*Practitioner.*)

Dr. L. E. Starr says that cantharidal blisters, moistened before application, with alcohol, will produce no strangury from the absorption of the active principle of the fly. Repeated trials with the above named result uniformly, lead him to give this fact to the profession.

4. *The Bromhydrate of Quinine.* GUBLER. (*Times and Gazette, Oct.* 9, 1875.)

Terminating his account of the Therapeutical Employment of Bromhydrate of Quinine (*Journal de Thérapeutique*, September 10), Prof. Gubler arrives at the following conclusions:—1. The bromhydrate corresponding to the sulphate of the same base is more soluble and more rich in alkaloid than the latter. 2. It possesses the physiological properties of the salts of quinine in general, and probably also the therapeutical virtues of its officinal congener. 3. Still, the action of the bromhydrate seems to differ from that of the sulphate of quinine, not only by the moderation of the symptoms of quinic intoxication (*ivresse*), but also by a marked tendency towards nervous sedation and hypnotism. 4. The qualities it thus possesses especially indicate its use in the treatment of the congestive and febrile affections of the nervous system—neuralgia-neuritis, irritative neuroses, encephalic hyperæmia, etc.,—in combating which it has already furnished excellent results. 5. The bromide has manifested remarkable power in a case of incoercible vomiting, and has frequently been of service in cases ordinarily amenable to the sulphate, as in visceral or articular fluxions, whether diathesic or not, rheumatismal or gouty, and in symptomatic fevers *à frigore*, etc. 6. This new medicine has been given in quantities of from six to fifteen grains per diem, in doses of three grains, administered sometimes in the form of pill, and at others of hypodermic injection. 7. Injected into the cellular tissue it has always proved absolutely inoffensive. In no case has the hypodermic injection of three grains of the bromhydrate (equivalent to about four and a half of the sulphate) been followed by the slightest inflammatory accident, neither redness nor tumefaction being visible next day around the seat of puncture. 8. This complete innocuity, joined to its greater solubility, constitute an incontestable superiority of this new combination of quinine, and recommend it wherever there is indication or necessity of administering quinine hypodermically.

5. *Cold Injections.* Dr. FELTZ. (*Lyon Médicale*, P. 3 & 4, 1875.)

The writer sums up his experiments with the following conclusions:

1st. The cold injection has a local and general physiological action.

2d. The local action consists of a sensation of coolness, followed by intestinal contraction.

3d. The general action produces a reduction of the pulse, diminution of animal temperature, and sedation of the nervous system; it abates the thirst, stimulates the appetite, and increases the secretions.

4th. This cooling, sedative and tonic action is uniform in its character for all injections, the temperature of which is below 38° (C.); but it is of greater intensity and more durable in proportion as it is colder and more abundant and often repeated.

5th. The therapeutic indications for cold clysters are numerous; it is useful, from its local action in diseases of the abdomen, and from its general action in febrile disorders. From this double action it is indicated and is successful, as a principal remedy, in typhoid fever.

6. *Use of Electricity for Hyperpyrexia in Typhoid Fever.* Gluk. (*Annali Univ. de Med.*, 1875.)

In thirty cases, where the temperature exceeded 102° F., the author used the following means: He applied the positive pole from a pile with constant current upon the third cervical vertebra, and the negative pole upon a level with the superior cervical ganglion of the great sympathetic. Without going into details, he says there was *always* a considerable lessening of the temperature.

7. *Electrolysis Applied to the Treatment of Various Tumors.* Luigi Ciniselli. (*Il Galvani*, 1874, Nos. 1, 2, 3.)

The author thus sums up the three modes of action of the electrolytic effects of the galvanic current: 1st, cauterization and destruction of the tissues; 2d, partial cauterization and modification of the involved pathologic tissues; 3d, modification of the tissues, without cauterization.

The tumors which have yielded the most happy results are the vascular tissues (angiectasiæ and cavernous angiomas). In more than 200 cases relating to tumors of this nature the result was favorable, save in one (pyohemia). Reaction was moderate, and the cure was often effected without suppuration.

In tumors composed of a liquid in a morbid or accidental cavity, the issue was less favorable.

Solid cystic tumors do not all behave in the same manner; still, most of the cystic tumors of the eyelid yield to the action of a current from sixteen to twenty-four elements of the pile of the author, in the space of one-third of a month, without leaving any traces.

Lipomas resist electrolysis. Fibrous tumors are easily removed by this means. The author recommends this method in sessile fibromas, situated in deep cavities, inaccessible to the knife (larynx, pharynx).

